# Probiotic Properties of *Lactobacillus plantarum* RYPR1 from an Indigenous Fermented Beverage Raabadi

**DOI:** 10.3389/fmicb.2016.01683

**Published:** 2016-10-21

**Authors:** Ruby Yadav, Anil K. Puniya, Pratyoosh Shukla

**Affiliations:** ^1^Enzyme Technology and Protein Bioinformatics Laboratory, Department of Microbiology, Maharshi Dayanand UniversityRohtak, India; ^2^Division of Dairy Microbiology, ICAR-National Dairy Research InstituteKarnal, India; ^3^College of Dairy Science and Technology, Guru Angad Dev Veterinary and Animal Sciences UniversityLudhiana, India

**Keywords:** *Lactobacillus plantarum*, probiotics, raabadi, hypocholesterolemic effect, cell surface hydrophobicity

## Abstract

Present study documents the potential probiotic *Lactobacillus* isolated from indigenous fermented beverage Raabadi, consumed during summers in Haryana and Rajasthan regions of India. A total of five Raabadi samples were collected aseptically and 54 isolates were purified using MRS medium. All the isolates were assessed for tolerance to low pH and bile salts. It was observed that out of 54 only 24 isolates could survive the simulated gastric conditions. These isolates were further evaluated *in vitro* for cell surface hydrophobicity, cell surface hydrophobicity, hypocholesteramic activity, anti-oxidative potential, BSH activity, antagonistic activity, and antibiotic resistance profile. In addition, the confirmation of phenol resistance was also done. On the basis of results obtained, the survival rate of isolates was noted and six isolates were finally selected for further studies. Among them *Lactobacillus plantarum* RYPR1 and RYPC7 showed good survival at pH 2 which shows good acid tolerance. Moreover, *L. plantarum* RYPR1 showed the highest hydrophobicity (79.13%) and represented the deconjugation of bile salts, which help in their adhesion to epithelial cells and colonization. Furthermore, RYPR1 also exhibited highest cholesterol reduction (59%) and subsequent analysis of results revealed that the above mentioned isolates further exhibit a good hypocholesterolemic effect and could be possibly used to prevent hypercholesterolemia. The present study divulges that *L. plantarum* RYPR1 has an excellent probiotic potential.

## Introduction

Raabadi is a popular indigenous fermented beverage in Haryana and Rajasthan regions of India. The ingredients of Raabadi are flour of barley and pearl millet, buttermilk, salt, black pepper, cumin seed powder, and dry green coriander. Since, it is a refreshing and cooling drink, therefore, mainly consumed during summers. The aim of present study is to evaluate the probiotic potential of Raabadi.

Probiotics is an emerging field for food manufacturers, predominantly in dairy food industry with remarkable growth potential (Mitropoulou et al., 2013). Probiotics are live microbial food supplements, which exert various health benefits on a host, when administrated in adequate amounts (Vinderola et al., 2008). These are generally referred as industrially important lactic acid bacteria (LAB) involved in fermenting dairy products, food, beverages, and produces lactic acid as a final product. Among these, the bacteria that exhibit good probiotic properties are *Lactobacillus* and *Bifidobacteria*. The success of probiotics in the market will depend on the effectiveness of the probiotic cultures used. Nowadays metabolic engineering for designing probiotics in food market have gained a lot of attention (Singh and Shukla, 2014). Genetic modification of microbes involves the introduction of desired genes that may have a positive impact on the food industry (Gupta and Shukla, 2015). Probiotics produce a diversity of compounds, i.e., exopolysaccharides, organic acids, bacteriocins, and antimicrobial compounds, etc. (Jacobsen et al., 1999). The bacteriocins and acids produced depress the growth of putrefactive microbes in the gut (Pessione, 2012).

An effective probiotic should be viable, safe, bile and gastric juices tolerant, able to survive through the gastrointestinal tract, adhere and colonize gut epithelial cells (Casey et al., 2004; Singh et al., 2011; Yadav and Shukla, 2015). Bacterial Adhesion to Hydrocarbons (BATH) was performed for studying their adherence to gut surfaces which enhance their interaction with the host (Kumar et al., 2012). Phenol tolerance is another important selection criterion, as gut bacteria can deaminate diet derived amino acids leading to the formation of phenolics. These bacteria confer a number of health benefits to the host, i.e., enhancement of immune function, lipid reduction, serum cholesterol reduction, anti-allergic, anticancer, antimicrobial, antioxidative properties etc. (Lee et al., 2014). Bacteriocins produced by probiotics have an inhibitory effect against pathogens. There are also evidences of their role in treating ulcerative colitis, necrotizing enterocolitis, pouchitis, acute infectious diarrhea, antibiotic-associated diarrhea, irritable bowel syndrome, and Crohn's disease (Chong, 2014). Probiotics have shown favorable properties in liver disease treatment, modulating permeability of the intestine, and inflammatory response. The supplementation of probiotic *Lactobacillus paracasei* results in decreasing *Enterococcus* spp. and *Enterobacteriaceae* that may promote the growth of intestinal beneficial microflora i.e., *Lactobacillus, Bifidobacteria* etc. (Chávez-Tapia et al., 2015). Furthermore, probiotics have the tendency to improve antioxidant activity and used as adjuvant in treating cancer, allergy, lactose intolerance, vaginosis, and *Helicobacter pylori* infection (Vieira et al., 2013; Touchefeu et al., 2014; Hill et al., 2014; McFarland, 2014; Narbona et al., 2014; Maldonado-Galdeano et al., 2015).

Recently few studies suggested that *L. plantarum* is a versatile and one of the main LAB due to its distinctive probiotic properties (Cammarota et al., 2009) and it can tolerate acidic and bile conditions and has antagonistic activity against intestinal pathogens (De Vries et al., 2006). Therefore, in the present work, we have studied the probiotic properties of Raabadi. The objective of this study was to evaluate the probiotic potential of *L. plantarum* isolated from Raabadi, for their possible use in the preparation of fermented drinks.

## Materials and methods

### Collection of samples, isolation, and purification of *Lactobacillus* isolates

Raabadi samples (05) were collected from different regions of Haryana, following standard microbiological protocols. For isolation of LAB, 1 ml of each sample was dissolved in De Man Rogose Sharpe (MRS) broth and incubated overnight at 37°C. Serial dilutions of inoculated broth up to 10^−6^ were made using normal saline and pour plated on MRS agar plates. The plates were incubated for 48 h at 37°C for bacterial growth and observed for the appearance of colonies. Morphologically different colonies were streaked on MRS plates. Working cultures were maintained on MRS agar slants at 4°C and sub-cultured every 4 weeks for further analysis (Goyal et al., 2013).

### Screening of probiotic properties of *Lactobacillus* isolates

#### Acid tolerance

The isolates were incubated overnight in MRS broth at 37°C. Actively grown cells were harvested by centrifugation (7000 rpm, 4°C, 10 min). The pH of MRS broth was adjusted at pH 2.0 with 1N HCl (Ramos et al., 2013). MRS broth adjusted to pH 6.5 was used as a control. Harvested cells were resuspended in MRS broth with acidic pH and incubated at 37°C. After a time interval of 0, 1, and 2 h samples were withdrawn and serially diluted in phosphate buffer saline (PBS). Samples were plated on MRS agar plates and incubated at 37°C for 48 h. Cell viability was assessed by the plate count method and the results were expressed as log cfu/ml.

#### Bile tolerance

Overnight precultures were harvested and resuspended in 5 ml of MRS medium supplemented with 0.3% Oxgall and without as control (Ramos et al., 2013). After inoculation, samples were incubated at 37°C. After a time interval of 0, 1, 2, and 3 h samples were withdrawn and serially diluted using normal saline. Viable cell colonies were enumerated at 0, 1, 2, and 3 h by plating 100 μl of cultures of appropriate dilutions onto MRS agar.

#### Antagonistic activity of isolates

Antimicrobial activity of isolates against pathogenic strains was assessed using agar well diffusion method (Ridwan et al., 2008). Test microorganisms were *Escherichia coli, Staphylococcus aureus, Pseudomonas aeruginosa* and *Salmonella albony*. The pathogen (100 μl) was added to soft agar, mixed and overlaid on Muller Hinton Agar (MHA). Wells were made on MHA plates using borer. A 100 μl of overnight grown *Lactobacillus* culture was poured into a well on plates. Plates were allowed to dry and incubated at 37°C for 24–48 h.

#### Antibiotic susceptibility

Various methods such as *E*-test agar dilution, agar disk diffusion, etc. of determining antibiotic susceptibility of LAB has been described by researchers (Klare et al., 2005; Zonenschain et al., 2009). The antibiotic susceptibility of *Lactobacillus* isolates was assessed on Mueller-Hinton agar (MHA) plates using antibiotic disc diffusion method (Singh et al., 2012). MHA plates were poured and allowed to solidify at room temperature. Freshly grown bacterial cultures (100 μl) were spread on MHA plates and allowed to dry. Antibiotic discs were placed on these plates and were incubated at 37°C for 2 days. The diameter of zone of inhibition was measured by using an antibiotic zone scale. The results obtained were expressed in terms of susceptibility, moderate susceptibility, or resistance. Results were compared with interpretative zone diameters described by Performance standards for Antimicrobial Disk Susceptibility tests (CLSI, 2007). Antibiotic susceptibility pattern of isolates was assessed using Penicillin G (10 μg), Ampicillin (10 μg), Streptomycin (10 μg), Tetracycline (30 μg), Chloramphenicol (30 μg), Nalidixic acid (30 μg), Kanamycin (30 μg), Azithromycin (15 μg), Gentamicin (10 μg), Ciprofloxacin (5 μg).

#### Resistance to phenol

Gut bacteria can deaminate aromatic amino acids, which are derived from dietary proteins and may lead to the formation of phenols. These phenol compounds can inhibit the growth of LAB. Therefore, resistance to phenol by probiotics is important for their survival in the gastrointestinal tract (Xanthopoulos et al., 1997). The overnight grown cultures were inoculated in MRS broth with 0.4% phenol. After 0 and 24 h intervals, cultures were spread on MRS agar plates using serial dilution method. Cell viability was enumerated using plate count.

#### Production of H_2_O_2_

Production of H_2_O_2_ was determined by spotting overnight grown cultures (10 μl) on MRS plates containing 2,2-azino-bis-3-ethylbenzothiazoline-6-sulphonic acid (ABTS) (0.5 mM) and 2 mg/L horseradish peroxidase. MRS agar plates were incubated for 48–72 h at 37°C under anaerobic conditions. The appearance of a blue halo around colonies considered as a positive test for H_2_O_2_.

#### Lysozyme resistance

Overnight grown cells of Lactobacilli isolates were centrifuged (7000 rpm, 10 min, 4°C). The cells were washed twice with PBS and pellets were re-suspended in Ringer solution. The suspension (10 μl) was then inoculated into sterile electrolyte solution (CaCl_2_ 0.22 g/l; NaCl 6.2 g/l; KCl 2.2 g/l; NaHCO_3_ 1.2 g/l) with 100 mg/l lysozyme. Inoculation of isolates in solution without lysozyme was taken as control. Samples were incubated at 37°C and after 2 h of incubation viable cell count was enumerated using plate count method (Zago et al., 2011).

#### Cell auto-aggregation

The auto aggregation ability of bacteria, maintains the bacterial population in the gut (Rickard et al., 2003). Cell auto-aggregation was tested using the method described by Tomas and Nader (2005). The cultures were allowed to grow in MRS broth for 16–18 h and harvested by centrifugation. Harvested cells were washed, re-suspended in PBS and adjusted to an absorbance of 0.5 at 600 nm. The suspension was incubated at 37°C for 2 h. One milliliter of upper phase was removed carefully to measure the absorbance at 600 nm. Cell auto aggregation was measured by decrease in absorbance and measured by using the following formula.

$$\begin{array}{l} \text{Percent auto-aggregation }=\text{ Initial OD }-\text{ final OD}/\;\\ \;{\text{Initial OD}}^{*}100 \end{array}$$

#### Cell surface hydrophobicity

Bacterial adhesion was determined to assess the adherence potential of microorganisms to surface hydrocarbons, which is a measure of adhesion to epithelial cells of the gut. The cultures were allowed to grow in MRS broth for 16–18 h and centrifuged. Pellets were washed twice with phosphate urea magnesium sulfate buffer. Pellets were re-suspended in buffer, vortex, and adjusted to absorbance 0.7–0.9 at 610 nm. The cell suspension (3.0 ml) was mixed with 1 ml of hydrocarbon (xylene) and incubated at 37°C for 1 h for aqueous and organic phase separation. The aqueous phase (1 ml) was carefully removed and absorbance was measured at 610 nm (A_1_). Percent hydrophobicity was measured by a decrease in absorbance and calculated using following formula (Lee et al., 2011).

$$\text{Percent hydrophobicity }=\;(1\;-{\text{A}}_{1}/{\text{A}}_{0})\;\times \;100$$

#### Bile salt hydrolase (BSH) activity

The BSH activity of selected isolates was determined as per Nguyen et al. (2007). For this, overnight grown cultures were streaked on MRS agar medium supplemented with 0.37 g/l CaCl_2_ and 0.5% (w/v) different bile salts (sodium tauroglycocholate); sodium taurocholate; sodium taurodeoxycholate. Plates were incubated at 37°C in an anaerobic jar for 2–3 days. BSH activity was indicated by observing precipitation zones of hydrolyzed salts around the colonies.

#### Response to simulated stomach duodenum passage (SSDP)

SSDP assay represents complete environment for the survival of LAB in the stomach and duodenum of human (Mathara et al., 2008). For this, MRS broth (pH 3.0), synthetic duodenum juice (Na_2_HCO_3_-6.4 g, KCl 0.239 g, NaCl-1.28 g, distilled water-1000 ml, pH-7.5), Oxgall solution (10 g/100 ml) were prepared. The 16–18 h grown cultures of all six isolates were inoculated in MRS broth. Samples were drawn after 0, 1 h and viable cell count were determined by the spread plate method. After 1 h incubation, 4 ml oxgall was added, followed by 17 ml duodenum juice. Samples were incubated at 37°C and withdrawn after 2, 3 h for viable cell count and percent survival was calculated after 3 h.

#### Safety aspects of selected strains

##### Hemolytic activity

Hemolytic activity was examined by streaking the overnight grown isolates on blood agar plates (7% v/v sheep blood). Plates were incubated at 37°C for 2–3 days and observed for the zone of hemolysis around colonies (Pieniz et al., 2014).

##### DNase activity

Isolates were streaked on DNase agar medium to check the production of DNase enzyme. Plates were incubated at 37°C for 2 days. Plates were observed for the zone of DNase activity. Clear and pinkish zone around colonies were considered as positive for DNase activity (Gupta and Malik, 2007).

#### *In vitro* cholesterol assimilation

For cholesterol assimilation analysis MRS Thio broth (supplemented with 0.2% bile salt) was prepared. Filter sterilized cholesterol solution was added to the broth and inoculated with 1% culture followed by anaerobic incubation at 37°C for 24 h. Supernatant was collected by centrifugation (5500 rpm, 7 min, 4°C). 0.5 ml of supernatant was added to 3 ml of 95% ethanol followed by 2 ml KOH and the mixture was heated at 60°C for 10 min. After cooling the mixture, 5 ml n-hexane and 3 ml distilled water was added and allowed it to stand for 15 min at room temperature for phase separation. Once the phase was separated, 2.5 ml of upper hexane layer was removed. Four milliliter of o-phthaladehyde reagent was added to each tube and allowed to stand for 10 min. Then 2 ml of concentrated sulphuric acid was added, mixed and allowed to stand for 10 min. Absorbance of un-inoculated and spent broth was measured at 550 nm. Percent reduction was determined by the following formula (Singh et al., 2012).

$$\begin{array}{l} Cholesterolremoval(\%)\;=\;(Chouninoculated-Choinoculated\;\\ \;\times 100/Chouninoculated) \end{array}$$

#### Antioxidative potential by ABTS (2,2 azino-bis 3 ethylbenzothiazoline-6-sulfonic acid) method

This method was based on principle of scavenging ABTS radicals by *Lactobacillus* cultures. Overnight grown cultures were centrifuged at 5000 rpm for 15 min. ABTS was dissolved in water and working solution (5 ml ABTS solution mixed with potassium persulphate) was prepared. Overnight incubation was done in dark bottles for the generation of ABTS radicals. An aliquot of 200 μl of this working solution was added to PBS (15 ml) and absorbance was set at 750 nm. Cell suspension (10 μl) was added to the above solution and mixed for 30 s. Absorbance was recorded for 5 min at interval of 30 s and percent inhibition of absorbance was calculated (Singh et al., 2012).

### Physiological and biochemical characterization of isolates

Selected isolates were examined for morphological and biochemical identification. Morphological identification of isolates was done microscopically by gram staining and negative staining. Cellular morphology and colony characteristics of isolates were also examined on MRS agar. Also, catalase and endospore test were performed. Only gram +ve, non-spore forming, catalase negative isolates were further identified. Biochemical tests such as nitrate reduction assay, IMViC and citrate utilization tests recommended in Bergey's Manual of Determinative Bacteriology were performed. Fermentation of different carbohydrates by isolates was also determined.

### Molecular identification

Isolates were selected on the basis of probiotic properties and genus was identified using *Lactobacillus* genus specific primers Lb1-F and Lb2-R which amplify a region of 250 bp corresponding with the 16S rRNA gene. Genotypic identification of selected isolates was determined by gene amplification using genus specific PCR. Genus specific primers consisted of forward primer (Lb1-F, 5′-AGAGTTTGATCATGGCTCAG-3′) and reverse primer (Lb2-R, 5′-CGGTATTAGCATCTGTTTCC-3′). A total of 25 μl reaction mixture was prepared for PCR reactions. The reaction mixture containing each dNTP (0.2 mM), 0.5 μl DNA template (50–100 ng), forward and reverse primer (10 pmol), 1 × PCR buffer and Taq polymerase (0.5 U). The optimum conditions for PCR involved initial denaturation step of 5 min at 95°C followed by 35 cycles denaturation for 1 min at 95°C, annealing for 1 min at 55°C, extension for 5 min at 72°C, final extension for 7 min at 72°C. PCR products were separated on 0.8% agarose gel and 1 × TAE buffer (pH 8) was prepared containing 2 μl ethidium bromide. A 5 μl of PCR product was loaded with 3 μl of loading dye. The best isolate among the six isolates selected on the basis of tested probiotic properties, was identified using 16S rRNA sequencing. The sequencing was performed using universal primer pA-F (5′-AGAGTTTGATCCTGGCTCAG-3′) and pH-R (5′AAGGAGGTGATCCAGCCGCA-3′). The PCR product was sent for commercial sequencing and the sequence obtained was compared using BLAST.

### Statistical analysis

All the experiments were performed in triplicates and the results were expressed as mean ± *SD* of triplicates. The mean and standard deviation (*SD*) were calculated by putting the values in various statistical analyses when required, using Microsoft R Excel 2007 Software Package, “Microsoft Corporation, (Redmond, WA, USA)” and SigmaPlot 10.

## Results

### Acid and bile tolerance

A total of 54 bacterial cultures were isolated and initially screened for acid and bile salt tolerance in which it was observed that out of 54 only 24 isolates showed survival. Data of six isolates which fulfilled all the tested selection criteria for a probiotic strain and showed good probiotic properties are presented here. Strains were assessed for acid tolerance at pH 2 by using viable cell count method (Table 1). Acid tolerance is an important selection criteria as a probiotic strain should be able to survive gastric acidic conditions. Bile salt tolerance is prerequisite for the metabolic activity and colonization of bacteria in the small intestine of the host (Havenaar et al., 1992). The survival percentage of bacteria was determined after exposure to 0.3% bile salt for 3 h (Table 2). After exposure to acid and bile for 3 h, strains showed good survival. Isolate RYPR1 and RYPR9 from Raabadi showed good survival after 3 h incubation.

**Table 1:** **Viable cell count of *Lactobacillus* strains at pH 2.0 (log cfu/ml)**.

**Isolates**	**Time**		
	**0 h**	**1 h**	**2 h**
RYPR1	9.24 ± 0.01	9.06 ± 0.18	9.04 ±.48
RYPR9	9.40 ± 0.04	9.37 ± 0.22	8.93 ± 0.47
RYPC5	9.37 ± 0.12	9.16 ± 0.39	8.67 ± 0.66
RYPC7	9.26 ± 0.01	9.21 ± 0.37	8.47 ± 0.88
RYPC15	9.31 ± 0.07	8.89 ± 0.88	7.96 ± 0.12
RYPK3	9.31 ± 0.22	8.67 ± 0.12	7.93 ± 0.11

*Values are mean ± SD of three independent determinations (n = 3) of each sample*.

**Table 2:** **Survival of isolates after 2 h at 0.3% bile concentration (log cfu/ml)**.

**Isolates**	**0 h**	**2 h**	**3 h**
RYPR1	7.69 ± 0.04	6.23 ± 0.11	6.19 ± 0.23
RYPR9	7.72 ± 0.09	6.27 ± 0.19	6.14 ± 0.25
RYPC5	7.66 ± 0.02	6.25 ± 0.04	6.09 ± 0.02
RYPC7	7.63 ± 0.07	6.04 ± 0.07	5.79 ± 0.04
RYPC15	7.70 ± 0.04	5.77 ± 0.02	5.26 ± 0.09
RYPK3	7.68 ± 0.01	6.12 ± 0.13	5.90 ± 0.11

*The mean of three values of each sample are presented along with ± SD*.

### Antagonistic activity of isolates

Isolates were tested for antimicrobial activity against common enteric pathogens (Table 3). On the basis of inhibition against all tested indicators and maximum zone of inhibition 6 isolates (i.e., RYPR1, RYPR9, RYPC5, RYPC7, RYPC15, RYPK3) were selected for screening of probiotic properties. Isolates showed inhibitory effect toward tested pathogens. Isolate RYPR1 showed good inhibition zones against *E. coli, S. aureus, P. aeruginosa*, and *S. albony*.

**Table 3:** **Antagonistic activity of isolates and zone of inhibition (ZOI) against tested pathogens**.

**Isolates**	**Tested bacterial strains (with ZOI in mm)**			
	***E. coli***	***S. aureus***	***S. albany***	***P. aeruginosa***
RYPR1	+	++	++	+++
RYPR9	+	++	+	+
RYPC5	+	+	++	++
RYPC7	+	++	+	+
RYPC15	−	+	+	+
RYPK3	−	+	−	−

*ZOI, Zone of inhibition, − no effect detected, + diameter of zone of inhibition less than 1 mm, ++ diameter of zone of inhibition between 2 and 5 mm, +++ diameter of zone of inhibition between 5 and 10 mm*.

### Antibiotic susceptibility

Selected isolates showed resistance to nalidixic acid, ciprofloxacin, and vancomycin. Also maximum susceptibility of all isolates was observed against ampicillin and erythromycin. Isolate RYPR1 showed resistance against maximum antibiotics tested (Table 4).

**Table 4:** **Antibiotic susceptibility pattern of selected isolates**.

**Antibiotics**	**Concentration (μg/disc)**	**RYPR1**	**RYPR9**	**RYPC5**	**RYPC7**	**RYPC15**	**RYPK3**
Tetracycline	30	R	S	S	S	S	S
Kanamycin	30	R	S	R	R	S	S
Nalidixic acid	30	R	R	R	R	R	R
Ampicillin	10	S	S	S	S	S	S
Gentamicin	10	R	R	R	R	S	S
Streptomycin	10	MS	S	S	S	S	S
Penicillin G	10	R	MS	R	MS	MS	MS
Erythromycin	15	S	S	S	S	S	S
Vancomycin	30	R	R	R	R	R	R
Ciprofloxacin	5	R	R	R	R	R	R

*R, resistant; S, sensitive; MS, moderately sensitive*.

### Resistance to phenol

Isolates that survived gastric juice conditions were further evaluated for phenol resistance, where these showed different degree of sensitivity toward phenol. Isolate RYPR1 showed good survival and was less sensitive toward phenol, whereas RYPK3 and RYPC15 were inhibited (Table 5).

**Table 5:** **Survival of isolates after 24 h at 0.4% phenol**.

**Isolates**	**Viable counts (log cfu/ml)**	
	**0 h**	**24 h**
RYPR1	7.41 ± 0.02	7.73 ± 0.3
RYPR9	7.37 ± 0.03	7.32 ± 0.05
RYPC5	7.39 ± 0.04	7.10 ± 0.09
RYPC7	7.42 ± 0.08	7.39 ± 0.07
RYPC15	7.43 ± 0.05	7.3 ± 0.01
RYPK3	7.45 ± 0.06	6.36 ± 0.03

*The mean of three values of each sample are presented along with ± SD*.

### H_2_O_2_ production

H_2_O_2_ production was evaluated by growing the isolates on MRS-ABTS plates. The bacterial isolates grown on plates varied in the intensity of color. Results showed that highest production of H_2_O_2_ was noted in RYPR1 and RYPR9 isolates. Lowest H_2_O_2_ production was shown by RYPC7. Isolates RYPC5, RYPC15, and RYPK3 did not exhibit the ability to produce H_2_O_2_. Since, H_2_O_2_ has inhibitory effects on other microorganisms, hence, it enhances the shelf life and safety of food products.

### Surface properties and lysozyme survival

Tested isolates were lysozyme resistant, where RYPR1 showed maximum survival of 96.69%, an average of log cfu/ml. Isolates were evaluated for cell surface properties toward hydrocarbon xylene. Isolates showed a variable degree of hydrophobicity. All isolates showed good surface hydrophobicity, but isolate RYPR1 showed maximum affinity toward xylene. Maximum hydrophobicity of isolates was RYPR1 (79.13%) followed by RYPR9 (77.82%). Cell aggregation involves interaction of surface components of a cell such as proteins, carbohydrates and lipoteichoic acid. The auto-aggregating cell clumps together and settled at the bottom of the tube, resulting in decreasing absorbance of suspension. Percent aggregation of selected isolates ranged from 53 to 81% (Table 6). Strain RYPR1 showed highest aggregation potential followed by RYPC5.

**Table 6:** **Lysozyme tolerance and surface properties of selected isolates**.

***Lactobacillus isolates***	**Lysozyme resistance (% survival)**	**% Aggregation**	**% Hydrophobicity**
RYPR1	96.69 ± 0.13	81.94 ± 0.13	79.13 ± 1.18
RYPR9	95.58 ± 0.01	62.93 ± 1.70	77.82 ± 0.31
RYPC5	81.04 ± 0.21	65.99 ± 1.18	66.78 ± 1.00
RYPC7	89.07 ± 0.70	55.02 ± 1.87	70.28 ± 0.31
RYPC15	53.45 ± 0.09	53.42 ± 2.50	70.0 ± 0.74
RYPK3	61.36 ± 0.07	61.04 ± 0.23	51.83 ± 0.04

*The mean of three values of each sample are presented along with ± SD*.

### BSH activity of isolates

Isolates were grown in the presence of high bile salt concentrations to assess their ability to hydrolyze bile salts. Plates were observed for precipitation zone and BSH positive strains were surrounded by a white precipitation zone. All strains were able to hydrolyse salts, which infer that isolates not only survived toxicity of these salts, but also carry out deconjugation of these salts and may help in their intestinal colonization (Table 7).

**Table 7:** **Bile salt hydrolase (BSH) activity of lactobacilli isolates**.

**Bile salt deconjugation**			
***Lactobacillus isolates***	TGC^*^	TC^*^	TDC^*^
RYPR1	++	+++	+++
RYPR9	++	++	++
RYPC5	+	++	++
RYPC7	++	++	++
RYPC15	++	+	++
RYPK3	+	+	++

−, no growth; +, growth only; ++, slight precipitation; +++, intense precipitation; TGC^*^, sodium tauroglycocholate; TC^*^, sodium taurocholate; TDC^*^, sodium taurodeoxycholate.

### Survival to SSDP

**Figure 1:**
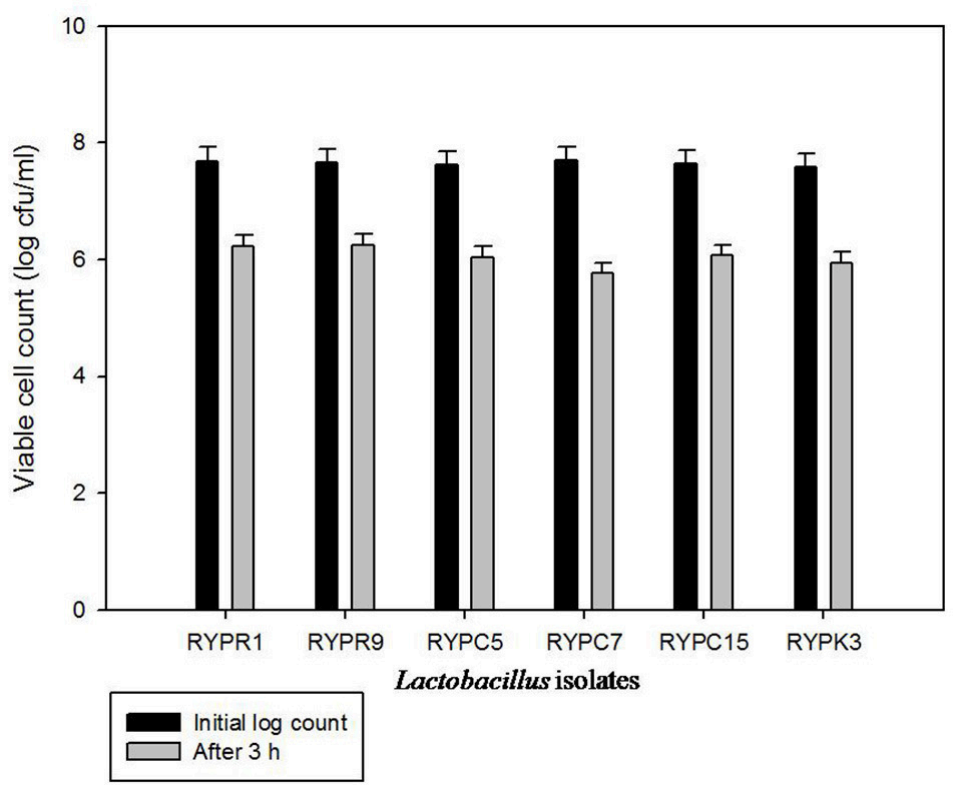
**Response of *Lactobacillus* isolates to simulated stomach duodenum passage**.

The test was carried out to evaluate the effect of all components (i.e., bile, low pH and duodenum juice) in a combined system. All isolates survived to this exposure, which suggested that isolates will be successful in reaching the intestine. Viable cell count of *Lactobacillus* isolates was calculated (Figure 1). The results showed that isolate RYPR9 showed a slight decrease in log count after 3 h exposure to ssdp and showed maximum survival of 81.59% followed by RYPR1 80.9%.

### Hemolytic and DNAse activities

A potential probiotic should be safe and non-pathogenic. Isolates were screened for DNAse and hemolytic activities and none of the strains exhibited hemolysis and DNAse activity. It means the selected isolates do not exhibit pathogenicity and are safe for consumption. All isolates selected for this study did not demonstrated hemolytic and DNAse activity as confirmed by zone of hemolytic and DNase activity.

### Cholesterol lowering ability

**Figure 2:**
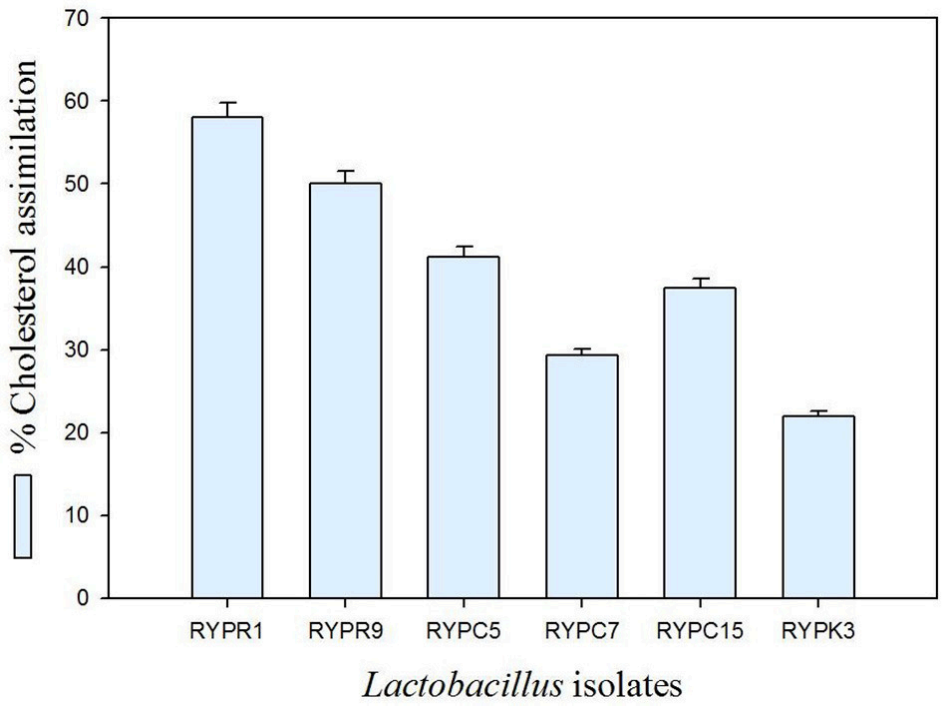
**Percent cholesterol assimilation of Lactobacilli isolates**. Values are mean ± *SD* of three different observations.

Selected isolates were screened for cholesterol assimilation and results obtained (Figure 2). Out of six isolates, RYPR1 showed the maximum cholesterol removal (59%) followed by RYPR9, RYPC5, RYPC15, RYPC7, RYPK3. Results showed that isolates with hypocholesterolemic effect could be used to prevent hypocholesterolemia.

### Antioxidative potential of *Lactobacillus* isolates

The Antioxidative potential of *Lactobacillus* isolates was investigated by the ABTS method. The results obtained by antioxidative analysis of selected isolates are presented in Table 8. Antioxidative potential of isolates ranged from 11.32 ± 0.16 to 78.53 ± 0.97%. All isolates showed inhibition of ABTS radicals except RYPC15. Highest antioxidative potential was observed for isolate RYPR1 (78.53 ± 0.97%) and RYPR9 (70.67 ± 0.57%).

**Table 8:** **Percent inhibition of oxidized radicals by isolates**.

**Isolates**	**% Inhibition**
RYPR1	78.53 ± 0.97
RYPR9	70.67 ± 0.57
RYPC5	50.73 ± 0.85
RYPC7	11.32 ± 0.16
RYPC15	–
RYPK3	54.88 ± 1.25

*The mean of three values of each sample are presented along with ± SD*.

### Physiological, biochemical, and molecular identification of isolates

All the isolates were Gram +ve, rod shaped, non-spore forming, catalase negative, and were able to ferment all the tested sugars (Table 9). On the basis of biochemical characterization, it was suggested that these isolates belonged to *Lactobacillus* genus according to Bergey's Manual of Bacteriology. Genus specific identification of isolates was also done, which further confirmed isolates as *Lactobacillus* genus. Isolate (RYPR1), which showed excellent probiotic properties were further identified by 16S rRNA sequencing and phylogenetic analysis. On the basis of BLAST results it has been found that the 16S rRNA sequence of above organism showed 99% similarity to *L. plantarum*. The sequence has been submitted to GenBank with accession number KX620369 (BankIt # 1939613).

**Table 9:** **Physiological and biochemical characteristics of isolates**.

**Parameters**	**Isolates**					
	**RYPR1**	**RYPR9**	**RYPC5**	**RYPC7**	**RYPC15**	**RYPK3**
Gram staining reaction	+ve, rods	+ve, rods	+ve, rods	+ve, rods	+ve, rods	+ve, rods
Endospore test	−	−	−	−	−	−
**BIOCHEMICAL CHARACTERIZATION**						
Catalase test	−	−	−	−	−	−
Oxidase test	−	−	−	−	−	−
Citrate utilization test	−	−	−	−	−	−
Methyl red test	−	−	−	−	−	−
Voges prausker test	−	−	−	−	−	−
Indole test	−	−	−	−	−	−
**CARBOHYDRATE FERMENTATION PATTERN**						
Glucose	+	+	+	+	+	+
Lactose	+	+	+	+	+	+
Sucrose	+	+	+	+	+	+
Xylose	+	+	+	+	+	+
Fructose	+	+	+	+	+	+
Ribose	+	+	+	+	+	+
Galactose	+	+	+	+	+	+
Dextrose	+	+	+	+	+	+
Maltose	+	+	+	+	+	+
Rhamnose	+	+	+	+	+	+
Mannitol	+	+	+	+	+	+
Trehalose	+	+	+	+	+	+

*+, Positive; −, Negative*.

## Discussion

The objective of the present study was to evaluate the probiotic potential of LAB isolated from Raabadi. Out of fifty four strains six showed good probiotic potential. Isolate RYPR1, which showed best probiotic potential was identified as *L. plantarum*. Several studies demonstrated the probiotic potential of *L. plantarum* from a wide variety of fermented foods (Pisano et al., 2008; Belviso et al., 2009). The preliminary screening involved behavior of strains toward acid and bile, which mimics the gastrointestinal tract conditions (Zago et al., 2011). Strains were further investigated for lysozyme resistance, as stress for microorganisms begins in saliva due to the presence of lysozyme. Selected isolates were able to survive lysozyme (100 mg/l), acid (pH 2), and bile (0.3%) (Mathara et al., 2008). Resistance to 0.4% phenol was further an important test conducted for checking the survival of *Lactobacillus* isolates under gastrointestinal conditions. Only *L. Plantarum* RYPR1 and RYPR9 were able to survive after an incubation period of 24 h. Vizoso-Pinto et al. (2006) reported that among 10 tested *L. plantarum* isolates, only four were able to tolerate phenol. Hydrogen peroxide production by *Lactobacillus* isolates was also of interest, as H_2_O_2_ may exhibit antimicrobial properties due to the presence of halides and peroxidases. Such type of reactions may lead to extension of shelf-life and enhancement of safety of food products. Vizoso-Pinto et al. (2006) also reported that out of 6 tested *L. plantarum* strains only two were able to produce H_2_O_2_. Using probiotics in food products may control food spoilage, extend shelf life and enhances food quality (Wei et al., 2006). *Lactobacillus* may inhibit pathogen colonization in gut, thus preventing various infections (Amin et al., 2011). Selected isolates were tested for antimicrobial activity against some common enteric pathogens. RYPC5 showed maximum inhibition zone against *E. coli*, followed by good inhibition zones against *S. aureus, P. aeruginosa, S. albony, L. plantarum* RYPR1 also showed good inhibition zones against *E. coli, S. aureus, P. aeruginosa*, and *S. albony*. A similar study was conducted by Ridwan et al. (2008) using agar well diffusion method. Kos et al. (2008) also reported antagonistic activity of probiotic strains against common pathogens *S. aureus, P. aeruginosa, E. coli, Y. enterocolitica, L. monocytogenes*. Another important probiotic property is BSH activity of isolates, which involves survival of bacteria under bile salts toxicity (Pisano et al., 2008). Isolates were grown in high bile salts concentrations; all strains were able to hydrolyse salts with RYPR1 *L. plantarum* showed maximum survival. Isolates were evaluated for cell surface properties and *Lactobacillus* strains showed a variable degree of hydrophobicity (Mathara et al., 2008). All isolates showed good surface hydrophobicity but isolate *L. plantarum* RYPR1 showed maximum affinity toward xylene (79.13%) than other isolates. Cell aggregation involves interaction of cellular surface components such as proteins, soluble proteins, carbohydrates, and lipoteichoic acid. *L. plantarum* RYPR1 showed highest aggregation potential followed by RYPR9. Selected *Lactobacillus* isolates were screened for cholesterol assimilation and *L. plantarum* RYPR1 showed maximum cholesterol removal (59%). High cholesterol level in blood could be a factor of risk in coronary heart disease. The observed hypocholesteromic effect among *Lactobacillus* strains could be due to their ability of deconjugation of bile salts into free radicals. As a result new bile acids from cholesterol start synthesizing. Therefore, total concentration of cholesterol reduces in body. It showed that *L. plantarum* RYPR1 exhibited hypocholesteromic effect and could possibly be used to prevent hypocholesterolemia.

## Conclusion

Probiotics have gained a lot of attention in food market due to the increased evidences of multidrug resistance among pathogens. So various users demand and require to use conventional or indigenous probiotics than long-lasting chemotherapeutics. The present study focuses on the expansion of selection of probiotic bacteria from indigenous fermented beverage raabadi. The results obtained from this study conclude that isolate *L. plantarum* RYPR1 showed good probiotic potential therefore, it could be used as starter culture while preparing probiotic based food products. Nevertheless, further exploration may be performed to confirm their potential health benefits and applications.

## Author contributions

All authors listed, have made substantial, direct and intellectual contribution to the work, and approved it for publication.

### Conflict of interest statement

The authors declare that the research was conducted in the absence of any commercial or financial relationships that could be construed as a potential conflict of interest.
